# Process evaluation of a randomised trial of a triple low-dose combination pill strategy to improve hypertension control: a qualitative study

**DOI:** 10.1136/bmjopen-2025-101689

**Published:** 2025-06-27

**Authors:** Abdul Salam, Tracey Laba, Rupasvi Dhurjati, Lakshmi K Josyula, Asita de Silva, Pavithra Godamunne, Rama Guggilla, Stephen Jan, Pallab Kumar Maulik, Nitish Naik, Anushka Patel, Arunasalam Pathmeswaran, Dorairaj Prabhakaran, Anthony Rodgers, Vanessa Selak, Ruth Webster (Griffiths)

**Affiliations:** 1 The George Institute for Global Health India, New Delhi, India; 2 University of New South Wales, Sydney, New South Wales, Australia; 3 Manipal Academy of Higher Education, Manipal, India; 4 The University of Sydney, Sydney, New South Wales, Australia; 5 The George Institute for Global Health India, Telangana, Hyderabad, India; 6 Indian Institute of Public Health, Hyderabad, Telangana, India; 7 University of Kelaniya Faculty of Medicine, Ragama, Western, Sri Lanka; 8 Department of Medical Education, Faculty of Medicine, University of Kelaniya, Colombo, Sri Lanka; 9 Uppsala University, Uppsala, Sweden; 10 All India Institute of Medical Sciences, New Delhi, India; 11 Department of Public Health, Faculty of Medicine, University of Kelaniya, Kelaniya, Sri Lanka; 12 Centre for Chronic Disease Control, New Delhi, India; 13 Public Health Foundation of India, New Delhi, India; 14 Epidemiology & Biostatistics, University of Auckland, Auckland, New Zealand; 15 The George Institute for Global Health, Sydney, New South Wales, Australia; 16 University of Technology Sydney Centre for Health Economics Research and Evaluation, Broadway, New South Wales, Australia

**Keywords:** Blood Pressure, Meta-Analysis, Hypertension, Systematic Review, CARDIOLOGY

## Abstract

**Background:**

High blood pressure (BP) is a significant global health issue, with many treated patients failing to achieve BP control. The Triple Pill vs Usual Care Management for Patients with Mild-to-Moderate Hypertension (TRIUMPH) trial evaluated the effectiveness, cost-effectiveness and acceptability of early use of low-dose triple fixed-dose combination of BP-lowering drugs (‘triple pill’) compared with usual care in the management of hypertension. The TRIUMPH trial showed superior BP control with the triple pill strategy compared with usual care. This process evaluation of the TRIUMPH trial aimed to explore the contextual factors that influenced the trial outcomes, implementation of the triple pill strategy, mechanisms of its effects and potential barriers and facilitators for implementing the triple pill strategy in routine practice.

**Methods:**

Guided by the UK Medical Research Council’s framework, semistructured interviews were conducted with 23 patients and 13 healthcare providers involved in the TRIUMPH trial. Data were analysed using the framework analysis method in NVivo.

**Results:**

Hypertension care in Sri Lanka was hindered by the absence of systematic screening and overcrowded public clinics. Despite free medication provision at public clinics, long waiting times and occasional stock-outs posed challenges. In the TRIUMPH trial, both intervention and usual care were delivered in the context of ‘better than usual’ care, including team-based management, reduced waiting times, monetary assistance for travel, routine adherence monitoring and intensive follow-up. The triple pill strategy provided a simplified regimen, better access to BP-lowering medications and better BP-lowering efficacy. Key barriers to implementation in routine practice included the triple pill’s large size, therapeutic inertia and restrictive regulatory policies regarding fixed-dose combinations.

**Conclusions:**

Implementation of the triple pill strategy into routine practice requires health system strengthening, provider training and supportive policy measures to replicate its effectiveness seen in the trial.

**Trial registration number:**

ACTRN12612001120864, SLCTR/2015/020.

**STRENGTHS AND LIMITATIONS OF THIS STUDY**

- Our study demonstrates methodological rigour by adhering to the Medical Research Council guidelines for evaluating complex interventions and by employing the six-stage framework analysis method.
- A limitation was the unavailability of the results of the trial at the time of the interviews, leading to lost opportunities for in-depth data collection on some unexpected findings (such as low up-titration despite falling short of blood pressure targets).
- Failure to interview pharmacists, site administrators and carers of patients, all of whom may have offered additional insights into the application, acceptability and potential scale-up of the triple pill, is another drawback.

## Introduction

 High blood pressure (BP) is a leading cause of mortality and morbidity worldwide. Despite the availability of numerous effective BP-lowering therapies, many patients with high BP remain untreated,^1 2^ and BP control in those treated is often suboptimal.^3 4^ Most patients with high BP need two or more BP-lowering medications to achieve guideline-recommended target BP.^5 6^ Recent guidelines recommend initiating BP-lowering treatment with combination therapy in most patients, making a shift away from the traditional ‘stepped-care’ approach of starting with a single medication, titrating to maximum dose and then adding a second medication.^5^ Seminal evidence synthesis of 354 randomised trials predicted that combining three antihypertensive medicines at quarter doses would lower systolic BP by about 20 mm Hg, providing rationale for early use of low-dose triple fixed-dose combination (FDC) of BP-lowering medicines (‘triple pill’) strategy.^7^

The TRIUMPH randomised open-label trial evaluated the triple pill strategy in 11 Sri Lankan hospital outpatient clinics. Patients with hypertension (either treatment-naïve or with BP uncontrolled on monotherapy) were randomised to once-daily low-dose triple pill (with a standard dose triple pill for up-titration if required) or usual care (in accordance with local hypertension care practice). Follow-up visits were conducted at 6 weeks, 12 weeks and 6 months postrandomisation. The primary outcome was the proportion of patients reaching target BP at 6 months.^8 9^ The trial demonstrated that the triple pill strategy significantly improves BP control compared with usual care (69.5% vs 55.3%; risk difference, 12.7% (95% CI 3.2% to 22.0%); p<0.001).

Sri Lanka has a high burden of untreated and uncontrolled hypertension,^10^ underscoring the implementation imperative for effective strategies like the triple pill strategy. However, like many low-middle income countries (LMICs), Sri Lanka poses challenges including constrained healthcare resources, non-availability of low-dose triple FDCs of antihypertensive medicines in the country, the current practice of predominantly using monotherapy as first-line treatment and significant therapeutic inertia.^11 12^ These contextual factors may hinder the implementation of the triple pill strategy despite the demonstrated effectiveness in a clinical trial. A process evaluation conducted alongside an RCT provides insights into why an intervention was effective—or why it was not—while also identifying barriers and facilitators to its implementation in clinical practice. Furthermore, a process evaluation can assist in developing hypotheses that will enable further analyses of data from the RCT.

Therefore, this process evaluation of the TRIUMPH aimed to explore the contextual factors that influenced the trial outcomes, implementation of the triple pill strategy, mechanisms of its effects and potential barriers and facilitators to implementing the intervention in routine clinical practice.

## Methods

### Theoretical framework and logic model

We designed the process evaluation in line with the UK Medical Research Council (MRC) guidance for process evaluation of complex interventions, which distinguishes three interacting domains—context, implementation and mechanisms of impact.^13 14^ Guided by this guidance, we built a logic model that linked the health problem, evidence, resources, core trial activities and anticipated outcomes of the TRIUMPH trial. The logic model is described in the process evaluation protocol manuscript, which was previously published^15^ and reproduced in the online supplemental figure S1. Semistructured interviews were conducted with trial patients and healthcare providers (HCPs) (physicians and their staff who participated in TRIUMPH (henceforth, providers)).

### Participant recruitment

From the TRIUMPH trial, a list of potential interviewees from both groups was prepared using maximum variation sampling considering participants’ age, sex, baseline BP and treatment, concomitant conditions (diabetes and cardiovascular disease (CVD)), income, lifestyle (physical activity, smoking and alcohol use) and adherence to BP-lowering treatment during trial follow-up. Typical case sampling was also used to reflect the average characteristics (age, BP and physical activity) of the trial patients (henceforth ‘patients’). Patients on this list were contacted by the trial staff before their end-of-trial follow-up visit and invited to participate in an interview during their visit. All the providers involved in TRIUMPH at each site were invited for the interviews through email. Providers who replied affirmatively and provided signed informed consent were scheduled for interview at a time convenient to them. Interviews were continued until thematic saturation was reached.

### Conduct of interviews

Interviews with providers were conducted by a male qualitative cardiovascular researcher from India (AS). Four female Sri Lankan science graduates, proficient in Sinhalese or Tamil, who were not involved in the TRIUMPH trial, were trained to conduct patient interviews. Patients who agreed to participate were introduced by the trial staff to the interviewers at the end-of-trial visit. Interviews were conducted at the trial site following the interview guides. Interview guides were mapped to the implementation, mechanism and context components of the MRC framework. Interviews were audio recorded while taking field notes. Only the interviewer(s) and the interviewee were present during the interview, unless the interviewee requested the presence of a companion (eg, a family member).

### Data management and analysis

#### Translation of interviews

Patient interviews (Sinhalese and Tamil) were transcribed verbatim in the original language and then translated into English by the interviewers following a standard transcription and translation guide. Provider interviews (all conducted in English) were transcribed by trained interns from India.

#### Analysis

Data were analysed by following the thematic analysis using the framework method.^16 17^ Initially, five interview transcripts were read thoroughly for familiarisation. Codes were developed using an inductive and deductive process by two authors (AS and TL) independently and in duplicate. Codes were first indexed deductively against the three MRC domains and the logic-model constructs, then refined inductively as new subthemes emerged. Data were coded by two researchers (AS and RD), independently in duplicate, in NVivo V.12. The researchers then developed the analytical framework from these codes and modified it subsequently as more interview transcripts were coded, and new codes and categories emerged. Two researchers (AS and RD) independently, and in duplicate, identified categories and codes relevant to the objectives of the study. Disagreements on the relevance of categories and codes were resolved in discussion with the research team. Data analysis was led by one (AS) of the two researchers involved in the trial’s implementation, while the other (RW), along with three other independent researchers (RD, LKJ and TL) of the trial, contributed to the analysis.

In Microsoft Excel, separate framework matrices were created for patients and providers, summarising and mapping codes. Themes were compared across interviewees, identifying patterns, including consonances and divergences, and generating memos describing the phenomena.

## Results

### Characteristics of interviewed patients and providers

Of the 700 patients in the TRIUMPH trial, 46 were identified as potential interviewees. One patient withdrew consent from the TRIUMPH trial after experiencing adverse events, and another declined to be interviewed. Between May 2017 and October 2017, a total of 23 patients (11 and 12 from the triple pill and usual care groups respectively) from all 11 sites were interviewed, with recruitment continuing until no new themes emerged (thematic saturation), consistent with empirical work showing that thematic saturation in homogeneous samples is typically reached within 9–17 interviews.^18^,^20^ Characteristics of the interviewed patients (table 1) were similar to those of all patients in TRIUMPH. We interviewed 13 providers (9 physicians including 6 cardiologists, 2 internal medicine specialists and 1 paediatrician and 4 research coordinators) at the end of the trial in December 2017. Provider recruitment was stopped after 13 interviews when the analysis team agreed that thematic saturation had been achieved.^18^,^20^ The interviews lasted an average of 17 min for patients and 30 min for providers.

**Table 1:** Summary of characteristics of interviewed trial patients

	Triple pill group	Usual care group	Total	TRIUMPH trial
Number of patients (%)	11 (47.8)	12 (51.2)	23	700
Females (%)	6 (54.5)	5 (41.7)	11 (47.8)	201 (57.5)
Age, years, mean (SD)	54.1 (13.2)	58.3 (7.0)	56.3 (10.4)	56.2 (11.0)
Diabetes mellitus (%)	4 (36.4)	3 (25.0)	7 (30.4)	110 (31.6)
Established CVD (%)	2 (18.2)	1 (8.3)	3 (13.0)	72 (10.3)
Systolic BP, mm Hg, mean, SD	149.7 (9.1)	152.4 (10.1)	151.2 (9.4)	154.2 (11.5)
Diastolic BP, mm Hg, mean, SD	88.0 (5.9)	92.5 (9.3)	90.3 (8.0)	89.8 (9.7)
Number on BP-lowering medications before trial	8 (72.7)	10 (83.3)	18 (78.3)	287 (41.0)
Current smokers (%)	0 (0.0)	3 (25.0)	3 (13.0)	36 (10.4)
Current drinkers (%)	0 (0.0)	2 (16.7)	2 (8.7)	43 (12.2)
Education: none/primary school	4 (36.4)	1 (8.3)	5 (21.7)	301 (43.0)

BP, blood pressure; CVD, cardiovascular disease; TRIUMPH, Triple Pill vs Usual Care Management for Patients with Mild-to-Moderate Hypertension.

**Figure 1:**
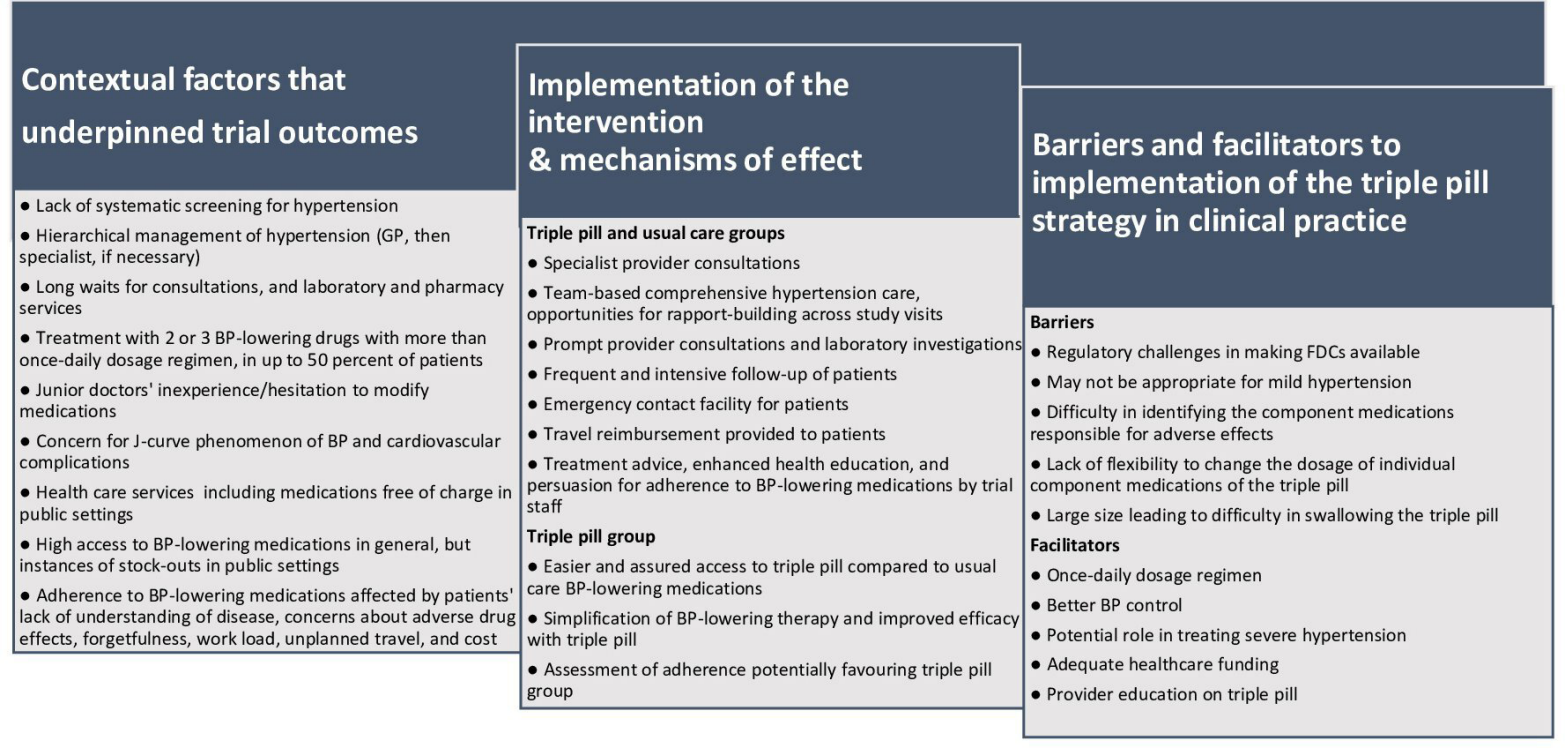
TRIUMPH trial context, implementation of the intervention and mechanisms of effects and barriers and facilitators to implementation of the triple pill strategy in clinical practice. BP, blood pressure; GP, general practitioner; TRIUMPH, Triple Pill vs Usual Care Management for Patients with Mild-to-Moderate Hypertension.

Thematic analysis identified four major themes, which were categorised as follows: contextual factors that influenced trial outcomes (healthcare settings), implementation of the intervention (trial setting), mechanisms of effect (factors likely contributing to the trial’s primary and secondary outcomes) and barriers and facilitators to implementation of the intervention (triple pill strategy) in routine clinical practice (figure 1). Findings are elaborated below with illustrative quotes from the interviews (online supplemental table S1).

### Contextual factors that influenced trial outcomes

#### Screening, diagnosis and awareness of hypertension

Systematic screening programmes were not in place for identifying individuals with hypertension. Providers in outpatient clinics were often reported to be rushed, leading to the likelihood of patients with high BP being overlooked and not referred to specialists when needed. Some patients were unaware of their elevated BP prior to participating in the trial as they had never had their BP checked before. A few patients said that they checked their BP frequently at home using their own BP monitors, while others stated that they had their BP measured only when they were unwell.

Provider 12: ‘In this hospital we don’t have a system to pick up the hypertension patients because we don’t have a screening process or nothing here. Accidentally/incidentally when they come here (they get diagnosed) …. We don’t have rules like after 40 [years of age] you have to check BP. We have programmes in Sri Lanka but not so effective. Not in our hospital. Usually, they do check-ups. We have a check-up unit, and they do annual check-ups’.

Provider 10: ‘They have to see 100 patients in 4 to 5 hours, for them it’s a matter of clearing the crowd. You know sometimes, marginally high BP, marginally tend to ignore. They might not even refer to us’.

Patient 2, triple pill group: ‘Previously I checked my blood pressure only when I fell sick or got fever and when I went for consultations’.

#### Care pathway for patients with hypertension

In the public healthcare centres where the TRIUMPH trial was conducted, a hierarchical approach to hypertension management was reported. General physicians (GPs) in the outpatient clinics initially managed the symptoms, with referrals to specialists for significant conditions such as severe hypertension and CVD.

Provider 5: ‘Usually when naïve patients, when they get the headache, usually go to GP first’.

Provider 2: ‘They may have features something related to the heart, not hypertension. But once in a blue moon you will get some patient with hypertension in addition to the heart problem’.

Long waiting times were reported by patients seeking consultations with GPs in outpatient clinics, where a first-come, first-served approach was followed. The procedures required to access services led to compounded delays due to the need for multiple appointments and movement between healthcare facilities. Laboratory investigations involved a sequence of steps, including filling in requisition forms, obtaining an appointment for sample collection, returning to collect the report and consulting providers in the outpatient clinic for review of the reports and advice.

Provider 13: ‘We just say clinic is from 8 to 12. So, they can come anytime. If they come early, they have to stay. We don’t give appointments.

Provider 5: ‘The blood reports, when patients come, we give them the requisition form. They have to walk to the out-patient department, and get a date, and then come back again on that date, and give the blood and get the report’.

#### BP-lowering medication use pattern

Providers reported that 10%–50% of their patients used combinations of two or three BP-lowering drugs. For a few patients, it was difficult to take multiple drugs every day, while others were accustomed to taking medications multiple times each day. A few patients were treatment-naïve, and one patient reported relying on indigenous medicines for hypertension control.

Patient 19, triple pill group: ‘Now I take the medications, take BP pills only in the morning. Before that, I was having them twice a day, in the morning and the evening’.

#### Patient follow-up and modification of therapy

Patients with hypertension were followed up by senior registrars every 3–12 months, and in parallel at a 1-month or 2-month interval by the GP at the outpatient clinic. Modification of BP-lowering medications was based on the condition of the patient and the experience of the provider: Junior physicians reportedly did not modify therapy, owing to their limited experience and preference to refer patients to seniors for therapy modification. Some providers deliberately avoided lowering patients’ BP to target for fear of hypotension and the ‘J curve’ phenomenon (potential inverse association between low BP and cardiovascular complications).

Provider 2: ‘Routine practice actually depends on the patient. Senior registrar usually sees the patients once in 3 months or 6 months and will be sent to the OPD clinic where they will be seen once a month or once in 2 months. Uptitrating the regimen depends on the level of the doctors. Junior doctor refers to the consultant whose appointment is obtained at least after a month during which the patient has to suffer with pressure. Junior level won’t change much frequently but senior level doctors adjust them because they have experience’.

Provider 3: ‘I don’t want to bring their blood pressure down because that deteriorates kidney functions and withdraw their blood pressure pills. I think this is the J effect phenomena’.

#### Access to BP-lowering medications

Access to BP-lowering medications in Sri Lanka was reportedly high, with medications provided free of charge at public hospital pharmacies. However, a high patient load at pharmacies sometimes results in long waiting times and occasional medication stock-outs, which lead some patients to purchase medications from private pharmacies, incurring out-of-pocket expenses. Most patients did not have health insurance but could access all services at public health centres free of charge.

Provider 1: ‘Reasonable access to the drugs in our part of the world is not so difficult’.

Patient 7, triple pill group: ‘If anything is required, I take them from government hospital. Only one month I had to buy one card of Aspirin from private pharmacy because it was out of stock here. I don’t have medication insurance’.

Patient 11, triple pill group: ‘I don’t like to hang around in that queue that turns like a reptile in the pharmacy. I get them from outside pharmacy’.

#### Patients’ adherence to BP-lowering medications

Some providers attributed poor adherence to BP-lowering medications to poverty and low literacy levels. They believed that patients with chronic conditions often became non-adherent due to a lack of understanding of their disease. Other reasons cited for poor adherence were misconceptions that the medications were harmful to health, and influence from complementary medicine practitioners who sometimes advised stopping allopathic treatments. Providers also reported ageing-related forgetfulness, adverse effects of medications, work pressure, polypharmacy and financial burden as additional factors contributing to low adherence. Some patients mentioned taking their BP-lowering medications only when their BP readings were high. Common reasons reported for non-adherence included work overload, medical emergencies, unplanned travel and forgetfulness.

Provider 7: ‘There are several practitioners in this country and these patients go to them and listen to them as well. When they go they record the BP, and say your BP is normal and you should stop your drug and see’.

Patient 3, triple pill group: ‘Sometimes I forget. Earlier I was having some memory issues. I still have forgetfulness’.

### Implementation of the intervention and mechanisms of effect

#### Better hypertension care within the trial

Patients in both the triple pill and usual care groups reported better than usual care throughout the trial, with treatment for their hypertension being provided by a dedicated team including specialist physicians. They underwent comprehensive evaluation and were accompanied by a trial staff member to their consultation where they received treatment advice and had their health-related questions answered. Patients were encouraged to contact the trial staff in case of medical emergencies and received telephonic reminders for follow-up visits, along with financial support for travel to and from the trial site. Additionally, patients did not have to wait in long queues for their consultations, biological sample collection for tests, and to collect the laboratory test reports, as these procedures were expedited at each study visit. Patients expressed a highly positive experience and gratitude for the courtesy, helpfulness and dedication of the trial staff. Overall, the hypertension care provided within the trial was substantially better than that available in routine practice (online supplemental table S2).

Patient 7, triple pill group: ‘Here we are given money and also medications. We are accompanied to the doctors. Generally, these activities do not happen in other hospitals. We ourselves have to do’.

Provider 10: ‘They are very courteous. That’s the main thing that lacks in most of our publicly run hospitals and not the private hospitals. Here I find there is a drastic change’.

Patient 2, triple pill group: ‘I never expected that they would take the blood specimens by themselves and deliver those reports to us. Usually that doesn’t happen in hospitals’.

Provider 6: ‘Patients were paid more attention, better explanations were given, got checked by a senior investigator. So maybe they had better control’.

#### Differential access to BP-lowering medications during the trial

Patients in the triple pill group received sufficient stock of medications at each study visit to last until the next visit, whereas the usual care patients had to collect their BP-lowering medications, monthly or bimonthly, from the hospital. Although medications were provided free of charge to all patients, usual care patients reported long waiting times at the hospital pharmacies and occasional stock-outs, which disrupted their medication schedules. One patient expressed concerns about the suboptimal packaging of medications provided by the hospital pharmacy, leading them to purchase from private pharmacies. Additionally, the provider noted that if a BP-lowering prescription was modified, the hospital pharmacy did not immediately dispense the revised prescription, causing delays in the implementation of therapy modifications.

Patient 20, usual care group: ‘Medications given from the hospital are without even a cover, just loose tablets right. Sometimes if we keep them in the pocket, they melt. Since such thing is not happening here (private pharmacies), I’m buying medications from outside’.

Provider 4: ‘One month of drugs were issued at a time. They had packets of various drugs and packets were given to the patient. In the middle of the study, changing the dose can be done but the dispensation does not take place’.

#### Follow-up during the trial

Patients’ attendance at the three trial follow-up visits was high and comparable between both groups. However, some patients were asked by the providers to attend additional follow-up visits for closer BP monitoring. Additionally, some patients made extra visits to the hospital pharmacy to collect medications for comorbidities, during which they could visit the trial site for BP measurement and medical advice. Among usual care group participants, an additional reason for visiting the trial site was the collection of BP-lowering medications from the site pharmacy between study visits. These visits included BP monitoring and management advice from site staff.

Provider 11: ‘If the patients are only taking the triple pill they are not coming for routine clinic visit to take medication. But the other patients if they are taking atorvastatin in addition to the triple pill they come back to collect it every month so, from those three months they had to come three times similar to the usual care patients’.

Provider 13: ‘Non-trial visits were similar to the normal visit. Only the thing is we don’t have to fill the eCRF, other than that everything is same, just like clinic’.

#### Simplification of BP-lowering therapy and improved efficacy with triple pill

Patients in the triple pill group took their medication once daily, whereas those in the usual care group followed varied regimens, taking medications once or multiple times per day based on their prescriptions. Initially, both patients and providers were apprehensive about the triple pill due to concerns about potential adverse effects. However, they later expressed satisfaction with its effectiveness in improving BP control. Some patients found it more convenient to take a single pill rather than multiple pills. For the providers’ perspective, prescribing the triple pill was straightforward, and they believed that its once daily dosing would also enhance adherence for patients.

Patient 18, triple pill group: ‘They told it at the very beginning, that it contains these constituents…. I could take all three types of pills together at once, rather than having them separately. I feel better, BP is normal. Now I take the BP pills only in the morning. Before that, I was having them twice a day. It is easy to swallow the pills because it is a capsule. I think this is better. It’s good, all medicines are together and even they are giving a low dose’.

Provider 3: ‘Although I lost apprehensions of starting treatment with three drugs, over the time, I still prefer to go slow, or in the sense go one after the other’.

For patients with uncontrolled BP, the trial protocol allowed for the triple pill to be up-titrated to a higher-dose version, and for the additional medications to be prescribed alongside either version of the triple pill. However, most providers noted that up-titration was rarely needed as the majority of the patients achieved BP control with low-dose triple pill combined with lifestyle modifications.

Provider 1: ‘The uptitration of the doses was not done very frequently as most of the patients were controlled on strength 1 [low dose of triple pill]’.

A few patients in the triple pill group believed that their BP was well controlled because the medication was manufactured in Australia. They perceived it to be of superior quality and, as a result, felt that non-pharmacological therapy was unnecessary.

Patient 1, triple pill group: ‘This is made in Australia. Not an Indian medicine. My blood pressure went down after taking the medications. Previously I was feeling sleepy at noon, but it is disappeared with start of this medication’.

#### Differential assessment of adherence to BP-lowering medications

Adherence to BP-lowering medications improved over the course of the trial, as trial staff consistently reinforced the importance of BP control and medication adherence during follow-up visits. Providers believed that offering healthcare advice to patients played a key role in improving adherence. In both groups, adherence to BP-lowering medications was assessed by using a 7-day recall question during trial follow-up visits, where patients reported the number of days they had taken their medication in the previous week. However, in the triple pill group, adherence was further monitored by counting the number of pills returned. If the number of pills returned deviated from the expected count, the trial staff discussed reasons for the discrepancy with the patient. This additional adherence monitoring in the triple pill group may have contributed to improved adherence and, consequently, better BP control. The overall emphasis on adherence in both groups likely had a positive impact on BP-lowering in the usual care group as well, as such reinforcement is generally less common in routine clinical care.

Patient 12, triple pill group: ‘Medicines were given and the trial staff checked whether I took the medicines regularly. Doctor asks me in every visit, whether I took the tablets, at what time I take the medicines, how many were taken, etc’.

Provider 12: ‘In triple pill group, we calculate dates from week 6 to week 12, the number of pills he has taken by counting which are remaining, and we take it to percent, and its above 80–85%, the compliance is satisfactory. If the compliance is less 90%, usually sub I [sub-Investigator] instruct them to take the triple pill accordingly. For usual care patients we don’t know. Some may or may not have taken medication seven days before the follow-up visit. But in the triple pill patients we know and advise them to take medication and explain possible complications*’*.

### Barriers and facilitators to implementing the triple pill strategy in clinical practice

#### Availability of FDCs of BP-lowering medications

Numerous BP-lowering medications were available in Sri Lanka for the treatment of hypertension. However, only a limited number of FDCs, consisting of just two BP-lowering medications, were available and these were relatively expensive. The use of FDCs was restricted due to the lack of approval from the regulatory authority, primarily driven by concerns about the adequacy of pharmacist training for dispensing such FDCs. However, these concerns were reportedly diminishing, and more FDCs were expected to receive approval in the future. Providers expressed hope that the results of the TRIUMPH trial would persuade the regulatory authorities to approve the triple pill.

Provider 7: ‘Regulatory authority is not happy to allow combinations. That is because they think the pharmacists are not trained enough to give the correct dose to the patient. I think that concept is going away’.

Provider 8: ‘I had lot of arguments, discussions and controversies about this with the authorities, and I have always been always positive for combination drugs, and so I have always been trying to persuade the authorities to get combination drug therapies, especially for hypertension. Availability of triple pill will be a breakthrough in the anti-hypertensive treatment’.

#### Acceptability of the triple pill

Both patients and providers agreed that making the triple pill available to the public at an affordable price would be highly beneficial. Most patients expressed willingness to recommend the triple pill to friends or family members with high BP, as they experienced effective BP control without adverse effects. Additionally, a few patients stated that they would purchase the triple pill regardless of the price, given the benefits they had experienced during the trial.

Patient 14, triple pill group: ‘Yes, recommending means, actually this is good. My wife also has hypertension. Some other medication is given to her. There are some other medications. So that means it is better to take this one medication rather than taking more’.

Patient 16, triple pill group: ‘It is a ‘divine medicine’. This pill, if it becomes popular among all patients, it’s a great service. If this medication is distributed among government hospitals and if this procedure continues in the future, it’s a great luck to our country’.

Patient 14, triple pill group: ‘I am willing to use triple pill post-trial even if I have to pay for it and even if it is costlier than other BP medications. It is better to pay for only one medicine rather than paying for three’.

Some providers expressed their willingness to prescribe the triple pill to their patients, post-trial, regardless of the trial outcomes, based on its ease of prescribing and their observations of its efficacy. They viewed FDCs as important facilitators of improved adherence to BP-lowering medications and believed that introducing the triple pill in Sri Lanka would be highly beneficial for hypertension treatment.

Provider 4: ‘Yes, I hope to see the results when it comes out. And even if it says similar to the existing treatment, even then I think it will be worthwhile trying it’.

#### Indication for, and limitations of, the triple pill

Providers were uncertain if the patients recruited in the trial, whose eligibility criteria included mild to moderate hypertension, accurately reflected the broader hypertensive population in clinical practice. In particular, they questioned whether patients at lower limits of mild hypertension, some of whom may have been classified as hypertensive based on BP readings at baseline, truly required treatment with the triple pill. Consequently, some providers believed that the triple pill might not be suitable as an initial therapy for mild hypertension in routine practice. Additionally, providers identified certain conditions where the triple pill might not be appropriate, including resistant hypertension, hypertensive emergencies and comorbidities such as renal impairment and diabetes. However, they suggested that the triple pill could be beneficial for treating severe hypertension, in addition to mild to moderate hypertension, as assessed in the trial.

Provider 6: ‘Now I would be little free or more lenient in using the combination in the patients with moderate hypertension, not the mild one as they can still get monotherapy, the severe ones definitely in any case can get multi drug’.

Provider 4: ‘It’s not very good for people with mild hypertension because people came back with a huge drop and complained faintish. In one patient we restarted triple pill and in a few we didn’t’.

Some providers expressed concerns about the up-titration of the triple pill for uncontrolled BP due to the lack of flexibility in adjusting the dosage of individual components. They suggested that the availability of a medium-strength triple pill would help address this limitation. Additionally, they noted that identifying which specific medication within the combination was responsible for adverse effects posed a challenge.

Provider 1: ‘When patients complain that legs are swollen, feeling itchy, then we may have to find which class drug, to find out you have to separately give all these three and see, taking off one by one which one is causing it. That’s of course a little bit challenge’.

Patients reported that the large size of the triple pill made it difficult to swallow. However, they were willing to take it since it required only once-daily dosing which they found convenient.

Patient 2, triple pill group: ‘To talk about experiences, that pill is a bit large in size because it has three drugs, but we can have it at once rather than taking three times’.

#### Healthcare funding and provider education

The implementation of the triple pill strategy in routine practice was perceived to be dependent on the budget allocations for public hospital services. Some providers believed that incorporating the triple pill into practice was feasible and emphasised the need for educating specialists on its benefits to facilitate its adoption.

Provider 15: ‘It is feasible to implement the same in general practice in Sri Lanka. I think our doctors should be educated. They should be given some knowledge on this. I think after the study, research should be available to every doctor to know about how this works. Most of them doesn’t know. So, they are scared to start combination therapy. So, after this study every medical person has to easily go through this’.

## Discussion

This process evaluation of the TRIUMPH trial identified that the trial was implemented in a context of ‘better than usual’ care, where patients received team-based care, increased provider attention, reduced waiting times for consultations and laboratory investigations, travel assistance and intensive health education and follow-up. Additionally, patients in the triple pill group benefited from simplified BP-lowering therapy through once-daily dosing and easier access to BP-lowering drugs. While opinions of the triple pill and its effectiveness were largely positive, several concerns emerged. These included the large pill size, uncertainty of its suitability for patients, and lack of a conducive regulatory environment for approving FDCs.

The TRIUMPH trial was designed as a pragmatic effectiveness trial comparing the triple pill strategy to usual care,^9^ to enhance the generalisability of the findings. However, ethical and regulatory requirements focusing on the efficacy outcomes led to both groups receiving ‘better the usual care’ making the usual care arm better than the routine care. Despite efforts to maintain non-differential implementation, some trial activities favoured the triple pill group.

Although trial participants received ‘better than usual’ care, BP control in the usual care group (56%),^9^ similar to a rural community study,^21^ suggests that better care did not significantly improve BP control. The improved BP control in the triple pill group (69% at 6 months) was likely due to the efficacy of the triple pill, facilitated by easier access to medications (dispensed at trial sites instead of hospital pharmacies), and close adherence monitoring through triple pill counts and querying patients in case of discrepancy. While both groups received adherence reinforcement, the TRIUMPH trial found no significant difference in adherence between the two groups.

The lack of difference in self-reported adherence was unexpected, given that simplified regimens are generally associated with improved adherence.^9^ The intense adherence monitoring applied to both groups as part of standard trial procedures may have diminished any additional adherence-promoting effect of the triple pill. Social desirability bias could also have influenced patient self-reports, as participants, gratified by the extra attention received during the trial, may have overstated adherence levels. Studies suggest that positive attitudes towards medications improve adherence, a finding reinforced by patients' appreciation of the trial staff’s education on BP control.^22^

Although both patients and providers were highly enthusiastic about the triple pill, BP control remained just under 70% in the triple pill group. Post hoc analysis revealed that while the triple pill helped overcome initial treatment inertia, providers were less likely to up-titrate therapy once patients were on the triple pill.^23^ This process evaluation highlights several provider-related factors contributing to this: the perception that most patients achieve BP control with low dose triple pills and lifestyle modifications; concerns about excessive BP reduction (J-curve phenomenon); a belief that the triple pill may not be suitable for mild hypertension; lack of flexibility in titrating individual components. These concerns, consistent with the challenges reported previously for implementing combination therapy,^24^ along with regulatory challenges for FDC approval, suggest that scaling up the triple pill strategy may be challenging despite its proven effectiveness.

### Strengths and limitations

This independent process evaluation included a diverse sample of patients interviewed at the end-of-trial follow-up, ensuring accurate recall of trial experiences while avoiding influence on trial performance. Patient interviews were conducted in local languages by trained interviewers, enhancing data reliability.

A key limitation was the unavailability of trial results at the time of interviews, which limited the opportunity to explore unexpected findings, such as low up-titration rates despite BP targets not being met. Additionally, pharmacists, site administrators and caregivers were not interviewed, missing potential insights into the practical application, acceptability and scalability of the triple pill.

This study focused on trial participants and providers to explore lived experiences within the trial. Future research should explore the perspectives of policy-makers, regulators and GPs, who could provide further insights into barriers and facilitators to integrating the triple pill strategy into routine clinical practice.

### Findings in the context of other evidence

A study by Perera *et al*^25^ on hypertension management in rural Sri Lanka highlighted similar challenges, including lack of systematic screening, poor adherence to BP-lowering medications, preference for complementary medicine, long waiting times and medication shortages—all of which were echoed in this evaluation.

Additionally, studies on cardiovascular polypills^26 27^ have shown that patients prefer FDCs due to the convenience of taking a single pill once daily, a finding strongly reinforced in our study.

### Implications for implementation scale-up and research

We suggest three priority actions for translating the findings of the TRIUMPH trial into routine hypertension care in Sri Lanka and similar LMICs. First, the main barrier cited was the absence of a low-dose triple FDC in Sri Lanka. Fast-track registration, immediate inclusion in the National List of Essential Medicines, and competitive tendering for an affordable generic product should be the first policy targets. Second, patients highly valued the short queues, scheduled appointments and team-based care received in the trial. Two elements are readily scalable: (1) task-sharing BP measurement, counselling and pill-counts with nurses or pharmacists and (2) converting walk-in clinics to timed appointments to reduce waiting. Both align with the WHO HEARTS technical package and could be piloted in hospitals. Third, concerns about the large size of the triple pill, therapeutic inertia due to fears of ‘overshooting’ BP targets, limited flexibility to adjust individual drugs, and lack of provider training were identified as implementation challenges. To address these, the triple pill should be developed in quarter, half and standard dose in small size formulations. In parallel, national hypertension guidelines should include a treatment protocol (pictorial titration algorithm) based on the triple pill strategy and be supported by brief continuous medical education modules that communicate the safety and efficacy of the triple pill strategy. Future research should explore the optimal implementation of the triple pill strategy in local contexts, with a focus on health system strengthening.

### Conclusions

The triple pill strategy significantly improved BP control among patients with mild to moderate hypertension compared with usual care, which itself exceeded routine care standards. The simplified therapy and superior BP control made the triple pill highly acceptable to both patients and providers.

For successful implementation in clinical practice, the triple pill strategy requires adequate funding, provider education and team-based care, alongside improvements in health system efficiency. However, to maximise its benefits, concerns regarding therapeutic inertia in up-titration and the large pill size must be effectively addressed.

## Supplementary material

10.1136/bmjopen-2025-101689 online supplemental file 1

## Data Availability

No data are available.
