# Supplementary material for: Process evaluation of a randomised trial of a triple low-dose combination pill strategy to improve hypertension control: a qualitative study
Source: BMJ Open. 2025 Jun 27;15(6):e101689. doi: 10.1136/bmjopen-2025-101689 (PMC12207132; doi:10.1136/bmjopen-2025-101689)
Supplement: online supplemental file 1 [file bmjopen-15-6-s001.docx]

Process evaluation of a randomised trial of a triple low-dose combination pill strategy to improve hypertension control: a qualitative study

Supplementary file

Figure S1: Logic Model


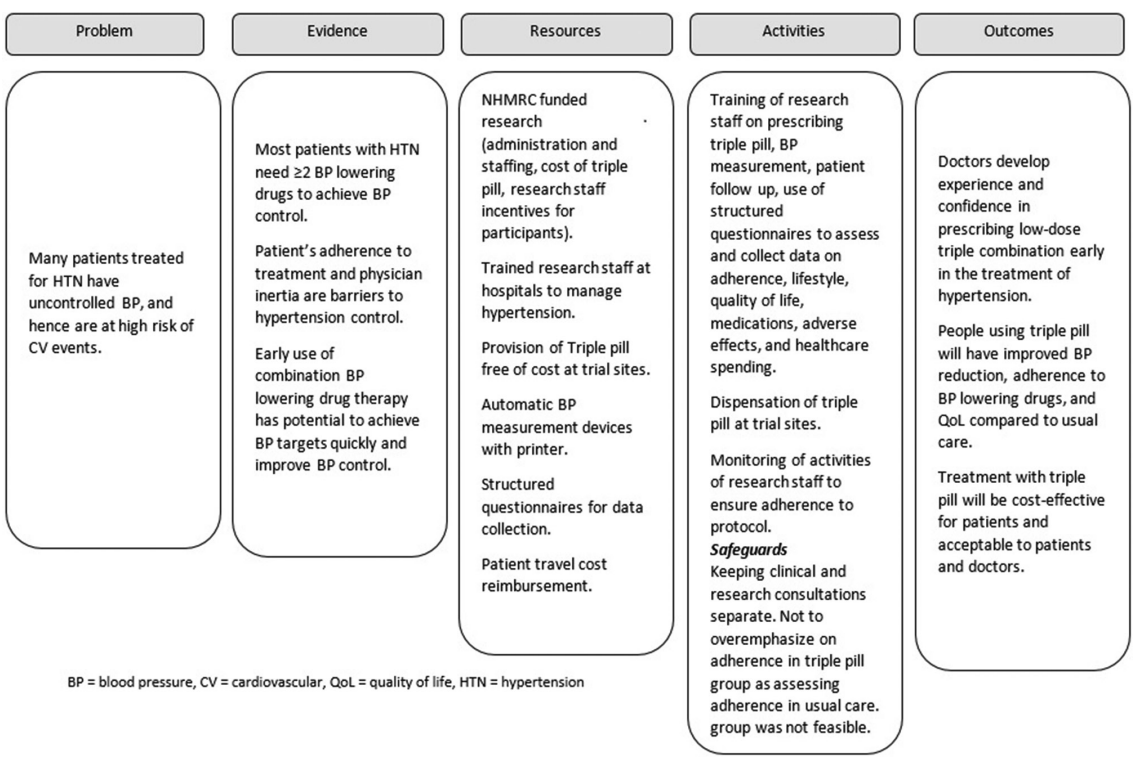


Figure reproduced from Salam A, et al. Process evaluation of a randomised controlled trial of a pharmacological strategy to improve hypertension control: protocol for a qualitative study. BMJ open. 2018 Aug 1;8(8):e022317, under the terms of the CC BY-NC 4.0 license.

Table S1: List of themes and sub-themes

| Themes | **Contextual factors that underpinned trial outcomes** | **Implementation of the intervention and mechanisms of effect** | **Barriers and facilitators to implementing the triple pill strategy in clinical practice** |
| --- | --- | --- | --- |
| Sub-themes | Screening, diagnosis, and awareness of hypertension | Better hypertension care within the trial | Availability of FDCs of BP-lowering medications |
|  | Care pathway for patients with hypertension | Differential access to BP-lowering medications during the trial | Acceptability of the triple pill |
|  | BP-lowering medication use pattern | Follow-up during the trial | Indication for, and limitations of, the triple pill |
|  | Patient follow-up and modification of therapy | Simplification of BP-lowering therapy and improved efficacy with triple pill | Healthcare funding and provider education |
|  | Access to BP-lowering medications | Differential assessment of adherence to BP-lowering medications | - |
|  | Patients’ adherence to BP-lowering medications | - | - |

Table S2: TRIUMPH trial context and implementation of the intervention

| **ACTIVITIES** | **ROUTINE PRACTICE** | **USUAL CARE GROUP** | **TRIPLE PILL GROUP** |
| --- | --- | --- | --- |
| ***Healthcare setting*** | | | |
| Treating doctor | General physicians | Cardiologist and/or cardiology registrars | Same as usual care |
| Consultation waiting time | Generally long due to  patient overload | Short | Same as usual care |
| Medications dispensed free  of charge to patients | Yes | Yes | Same as usual care |
| Patient-provider relationship | May be different healthcare providers at  different visits | Same team of healthcare providers at different visits allowing for rapport-  building | Same as usual care |
| Laboratory investigations | May/may not be free of charge to patients  Reports are to be collected at the lab  May not be available on time | Samples collected at study visit and processed at National Accreditation Board for Testing & Calibration Laboratories accredited lab  Free of charge to patients  Reports made available quickly to providers and patients | Same as usual care |
| Travel cost provided to  patients | No | Yes | Same as usual care |
| ***BP measurement, treatment and follow-up*** | | | |
| BP measurement devices | Manual mercury or  aneroid | Automatic | Same as usual care |
| Patient load influencing  quality of care | Yes | No | Same as usual care |

| Availability of antihypertensive  medications | Intermittent shortage | Shortage once or twice during the trial | No shortage |
| --- | --- | --- | --- |
| Medication dispensing | From hospital pharmacy Monthly supply  Often long waiting in the queue for refilling | From hospital pharmacy Monthly supply  Often long waiting in the queue for refilling | From the trial site  Supply to last till the next scheduled visit (6 weeks, 3 months, 6 months)  Patients receiving medications other than triple pill had to visit every month to receive those medications |
| Assessing adherence to medications | No process in place  Casual enquiry if patients are taking prescribed medications | Patient self-reported 7-day recall for adherence in the week prior to the follow- up visit | Patient self-reported 7-day recall for adherence in the week prior to the follow-up visit, and pill counting |
| Scheduled follow-up | Depends on patient’s  condition | 6 weeks, 3 months, 6 months | Same as usual care |
| Additional visits | Visits to hospital anytime if required | Visits to hospital anytime, if required  Visits for refilling prescriptions every month  Visits for trial follow-up  Additional visits for BP monitoring in some patients | Visits to hospital anytime if required Visits for trial follow-up  Additional visits for BP monitoring in some patients, if required |
| Telephone calls to remind the patient regarding  follow- up visit | No | Yes | Same as usual care |
| Comprehensive treatment  advice | May/may not be given. | Yes | Same as usual care |

| Easy access to a healthcare provider, in-person or  phone | No | Yes | Same as usual care |
| --- | --- | --- | --- |
