# Supplementary material for: Process evaluation of a randomised trial of a triple low-dose combination pill strategy to improve hypertension control: a qualitative study
Source: BMJ Open. 2025 Jun 27;15(6):e101689. doi: 10.1136/bmjopen-2025-101689 (PMC12207132; doi:10.1136/bmjopen-2025-101689)
Supplement: Supplementary file 2 [file bmjopen-2025-101689.reviewer_comments.pdf]

PEER REVIEW HISTORY

BMJ Open publishes all reviews undertaken for accepted manuscripts. Reviewers are asked to complete a checklist review form and are provided with free text boxes to elaborate on their assessment. These free text comments are reproduced below.

This paper was submitted to another journal from BMJ but declined for publication following peer review. The authors addressed the reviewers' comments and submitted the revised paper to BMJ Open. The paper was subsequently accepted for publication at BMJ Open.

ARTICLE DETAILS

Title (Provisional)

Process evaluation of a randomised trial of a triple low-dose combination pill strategy to improve hypertension control: a qualitative study

Authors

Salam, Abdul; Laba, Tracey; Dhurjati, Rupasvi; Josyula, Lakshmi K; de Silva, H Asita; Godamunne, Pavithra; Guggilla, Rama; Jan, Stephen; Maulik, Pallab Kumar; Naik, Nitish; Patel, Anushka; Pathmeswaran, Arunasalam; Prabhakaran, Dorairaj; Rodgers, Anthony; Selak, Vanessa; Webster (Griffiths), Ruth

---

VERSION 1 - REVIEW

**Reviewer**	**1**
**Name**	**Renna, Nicolas**
**Affiliation**	**Hospital Español de Mendoza, IMBECU-CONICET**
**Date**	**05-Sep-2024**
**COI**	**None**

Grammar Errors: The manuscript contains several grammatical errors throughout the text. It is advisable to conduct a thorough proofreading to rectify these errors and ensure clarity and coherence in the presentation of ideas.
Statistical Adequacy of Sample Size: While the article mentions the number of patients and providers interviewed (n=23 patients, n=13 providers), it is important to explicitly address whether this sample size is statistically adequate for drawing reliable conclusions. Consider discussing the rationale behind the chosen sample size and whether it provides sufficient power to detect meaningful differences or patterns in the data.
Placement of Results Paragraph in Methodology: The paragraph that initiates the discussion of results appears in the methodology section. To enhance the structural coherence of the manuscript, it is recommended to relocate this paragraph to the results section, where it

logically belongs.
Inclusion of Summary Table: Incorporating a summary table to encapsulate the key findings in a more concise and visually accessible format would greatly enhance the clarity and comprehensibility of the results. This table could provide a comprehensive overview of the main outcomes and comparisons, facilitating a quick understanding of the study's findings.
Redundancy in Results Section: The results section repetitively describes aspects of the methodology, leading to redundancy and potential confusion for the reader. Consider revising the results section to focus solely on presenting and interpreting the study findings, while transferring methodological details to the methodology section for clarity and coherence.

**Reviewer**	**2**
**Name**	**Sukonthasarn , Apichard**
**Affiliation**	**Chiang Mai University**
**Date**	**05-Sep-2024**
**COI**	**None**

In 4.1 Strengths and limitations, the authors mentioned that large numbers of patients were interviewed. However only 23 were interviewed out of 700. They should clarify statistically that this number was large enough to represent the whole studied group.

**Reviewer**	**3**
**Name**	**Gooden, Tiffany**
**Affiliation**	**University of Birmingham College of Medical and Dental Sciences**
**Date**	**05-Sep-2024**
**COI**	**None**

I congratulate the authors on completing a large and really important piece of work. A trial such as the TRIUMPH trial will undoubtedly benefit from a thorough process evaluation. However, I do think the manuscript is in need of major rework and improvements to the English throughout. The methods of the process evaluation is not very clear and the scientific merit of the analysis cannot be determined. For instance, a major weakness in the methodology is that you’ve mentioned using the MRC framework but it’s not explained how you used and applied the framework, what the logic model was, what kind of analysis was done, how your analyses answered your specific research question etc. I’ve attached specific comments for consideration.

Abstract

- It's best to define all acronyms when first used (e.g. BP)

- First sentence is quite long, making it difficult to follow.

- I'm not sure if the abstract layout follows the journal's guidance. It's difficult to understand what part of the abstract relates to the background, methods, results and conclusions. Would suggest amending organising this better by putting in labels/headers (check the journal's guidelines for specific instructions)

- The specific aim of the research is not entirely clear – I think it's because the TRIUMPH trial is not entirely clear – was it to test the effectiveness of the 3-pill strategy and the process evaluation was to evaluate the implementation of this? If so, I'm not sure why part of the process evaluation would include 'to understand hypertension care'? Surely you would aim for this before the trial?

- I won't comment more on the abstract but overall, it needs a lot of work and needs to be re-written.

Introduction

- The introduction is weak. Missing is any mention of existing evidence, how implementation of a triple pill strategy may or may not be challenging in Sri Lanka, how this may compare to other settings etc.

Methods

- Have a check of journal instructions but I would expect the trial registration number to be listed somewhere.

- Line 67 – gender or sex? It's likely the latter.

- Were all healthcare providers invited to participate or just some etc – more is needed on how they were recruited and selected.

- Line 73 – you say the interview was a male qualitative cardiovascular researcher but then line 77 says four female science graduates conducted the interviews. Better explanation of everyone's role is needed.

- If the topic guide was created based on the logic model, it would be useful to see the logic model – is this published elsewhere? If so, this should explicitly be referred to but if not, maybe think about adding it to the paper.

- Lines 97-99 – it sounds like 5 people in total were involved in the analysis, but who did what? How did this work in practice?

- Was the MRC framework used in the analysis? If so how? It's not clear what kind of analysis was done – was it thematic, content etc. Did you use induction or deduction analysis etc.

Results

- Line 107 – what was the rationale and aim for including patients from the control/usual care group? This wasn't really mentioned in the methods.

- Line 110 – how are research coordinators ‘providers’? More needs explaining on this in the methods.

- The average length of the interviews is concerningly low – how confident were you that you gathered all the necessary and relevant information? Definitely something to mention as a limitation.

- Table 1 looks more like presenting baseline characteristics of a quantitative study. Would suggest changing to remove proportions and also provide information on patients and healthcare providers separately.

- It’s not clear what exactly your themes are and whether there are sub-themes. Is ‘healthcare setting’ your first theme? A coding tree would be helpful and I suggest adding this in.

- Table 2 is not clear at all, I’m not sure what it’s trying to say or refer to.

- All direct quotes should be quotations

- Part of the results doesn’t seem directly related to the processes of the trial – e.g. under 3.2.1 you talk about the process of screening but was this part of the trial? It’s also not clear whether the information being reported is directly from the participants or is it an interpretation of the interview data/what participants said. This distinction is important. This all could be better understood with a clearer methods section.

Discussion

- I feel the discussion is overall weak. It seems more to do with the trial results and not the results of the process evaluation. Can you provide recommendations based on your results for future implementation and scale up?

VERSION 1 - AUTHOR RESPONSE

Reviewer: 1
Dr. Nicolas Renna, Hospital Español de Mendoza, IMBECU-CONICET
Comments to the Author:
Recommendation for Major Revisions

1. Grammar Errors: The manuscript contains several grammatical errors throughout the text. It is advisable to conduct a thorough proofreading to rectify these errors and ensure clarity and coherence in the presentation of ideas.

Thank you for pointing out the grammatical issues in the manuscript. We have carefully reviewed and thoroughly proofread the text to correct any grammatical errors and improve the clarity and coherence of the presentation.

2. Statistical Adequacy of Sample Size: While the article mentions the number of patients and providers interviewed (n=23 patients, n=13 providers), it is important to explicitly address whether this sample size is statistically adequate for drawing reliable conclusions. Consider discussing the rationale behind the chosen sample size and whether it provides sufficient power to detect meaningful differences or patterns in the data.

Thank you for your comment regarding the adequacy of the sample size. In qualitative research, the concept of sample size is not determined by significance levels (Type I error) or statistical power (Type II error). Instead, it is commonly based on thematic/theoretical saturation [1].

We ensured the sample size was methodologically robust by adhering to established principles of qualitative research, as detailed below:

1. **Rationale for Sample Size:**
   We employed maximum variation and typical case sampling techniques to ensure a diverse range of perspectives from both patients and providers. This approach allowed us to capture a broad spectrum of experiences, enhancing the richness and credibility of the data.

2. **Saturation Principle:**
   The sample size was determined based on thematic saturation, a widely accepted criterion in qualitative research. Thematic saturation occurs when no new themes or patterns emerge from subsequent interviews, indicating that the data is sufficiently comprehensive to address the research objectives (Ref). This was achieved in our study, as described in the Methods section.

3. **Methodological Justification:**
   The chosen sample size (23 patients, 13 providers) aligns with standards in similar process evaluation studies in healthcare research [2,3,4]. It provided a manageable yet sufficiently diverse and rich dataset for conducting a rigorous framework analysis, as outlined in the Methods section.

To address your comment, we have added text to the Results section clarifying the adequacy of the sample size based on thematic saturation and the methodological rationale outlined above.

3. Placement of Results Paragraph in Methodology: The paragraph that initiates the discussion of results appears in the methodology section. To enhance the structural coherence of the manuscript, it is recommended to relocate this paragraph to the results section, where it logically belongs.

Thank you for your observation. Upon reviewing the manuscript, we could not identify a paragraph in the Methods section that initiates the discussion of results. The Methods section is limited to describing the study design, data collection, and analysis processes, without including any result-oriented content.

To address your comment, we carefully re-examined both the Methods and Results sections and confirmed that all results are appropriately placed within the Results section. Additionally, we adhered to the “Consolidated criteria for reporting qualitative research (COREQ): a 32-item checklist for interviews and focus groups” [5] to ensure all relevant information is included and presented in proper order

4. Inclusion of Summary Table: Incorporating a summary table to encapsulate the key findings in a more concise and visually accessible format would greatly enhance the clarity and comprehensibility of the results. This table could provide a comprehensive overview of the main outcomes and comparisons, facilitating a quick understanding of the study's findings.
Thank you for your suggestion to include a summary table to encapsulate the key findings. While tables are often appropriate for summarising numerical data in quantitative studies, qualitative

research focuses on capturing rich, descriptive data that highlights the depth and nuances of participants' experiences.

We have **Table 2**, which provides a summary of the trial context and implementation of the intervention, including key elements such as healthcare settings, patient-provider interactions, medication availability, adherence assessments, and follow-up procedures. The thematic findings are elaborated in the text with supporting quotes to provide essential context and meaning, which may not be effectively conveyed in a tabular format.

5. Redundancy in Results Section: The results section repetitively describes aspects of the methodology, leading to redundancy and potential confusion for the reader. Consider revising the results section to focus solely on presenting and interpreting the study findings, while transferring methodological details to the methodology section for clarity and coherence.

Thank you for your comment regarding redundancy in the Results section. We reviewed the section carefully and found that the description of the trial's implementation, which you may be referring to, is included intentionally. This content provides essential context for comparing interviewees' views on usual care within versus outside the trial. Without this background, it would be challenging for readers to fully understand the findings.

To ensure clarity and reduce potential overlap, we have revisited the Results section and ensured that all methodological details not directly tied to the interpretation of findings have been retained in the Methods section. We believe this approach strikes the right balance between providing necessary context and avoiding redundancy.

Reviewer: 2
Dr. Apichard Sukonthasarn, Chiang Mai University
Comments to the Author:
In 4.1 Strengths and limitations, the authors mentioned that large numbers of patients were interviewed. However, only 23 were interviewed out of 700. They should clarify statistically that this number was large enough to represent the whole studied group.

Thank you for your observation. We acknowledge that describing the sample as "large numbers of patients were interviewed" was not appropriate, and we have revised the text to state that a diverse sample of patients and providers was interviewed.

Regarding the statistical sufficiency, please refer to the response for a similar comment from the first reviewer (First reviewer- 2nd question).

Reviewer: 3
Ms. Tiffany Gooden, University of Birmingham College of Medical and Dental Sciences
Comments to the Author:
I congratulate the authors on completing a large and really important piece of work. A trial such as the TRIUMPH trial will undoubtedly benefit from a thorough process evaluation. However, I do think the manuscript is in need of major rework and improvements to the English throughout. The methods of the process evaluation is not very clear and the scientific merit of the analysis cannot be determined. For instance, a major weakness in the methodology is that you've mentioned using the

MRC framework but it's not explained how you used and applied the framework, what the logic model was, what kind of analysis was done, how your analyses answered your specific research question etc. I've attached specific comments for consideration.

In response to your comments, we have undertaken the following actions:

1. **Clarification of the MRC Framework and Logic Model:**
   We have expanded the Methods section to clearly describe how the MRC framework was applied in this process evaluation. Specifically, we have detailed the components of the framework that guided our approach, including the development and application of the logic model. The logic model has been described to illustrate the relationships between the health problem, intervention activities, and expected outcomes.

2. **Description of the Analysis Process:**
   We have revised the text to provide a more comprehensive description of the qualitative analysis process, including how the framework method was applied to organise and interpret the data. This revision also highlights how the analyses were designed to address the specific research questions and explore the barriers, facilitators, and contextual factors influencing the implementation of the triple pill strategy.

3. **Improvement of English Language and Clarity:**
   We have thoroughly reviewed and revised the manuscript to improve the quality of the language and ensure clarity and coherence in presenting the methods and findings.

Commented [RD1]: Consider deleting this if we are satisfied with specific comments below.

Abstract

- It's best to define all acronyms when first used (e.g. BP)

We have made changes wherever required.

- First sentence is quite long, making it difficult to follow.

We have revised the text for clarity.

- I'm not sure if the abstract layout follows the journal's guidance. It's difficult to understand what part of the abstract relates to the background, methods, results and conclusions. Would suggest amending organising this better by putting in labels/headers (check the journal's guidelines for specific instructions)

We have added the sub-headings as per the journal's instructions.

- The specific aim of the research is not entirely clear – I think it's because the TRIUMPH trial is not entirely clear – was it to test the effectiveness of the 3-pill strategy and the process evaluation was to evaluate the implementation of this? If so, I'm not sure why part of the process evaluation would include 'to understand hypertension care'? Surely you would aim for this before the trial?

- I won't comment more on the abstract but overall, it needs a lot of work and needs to be re-written.

We thank the reviewer for their detailed comments on the Abstract. We acknowledge that there was significant room for improvement. We have substantially revised the Abstract to make it more

structured, covering the Background, Methods, Results, and Conclusion, while also improving the grammar and clarity.

Introduction

- The introduction is weak. Missing is any mention of existing evidence, how implementation of a triple pill strategy may or may not be challenging in Sri Lanka, how this may compare to other settings etc.

Methods

- Have a check of journal instructions but I would expect the trial registration number to be listed somewhere.

Thanks. We have added the trial registration number.

- Line 67 – gender or sex? It's likely the latter.

We confirm it was sex, which is what we have used.

- Were all healthcare providers invited to participate or just some etc – more is needed on how they were recruited and selected.

We have now added text to clarify how healthcare providers were recruited.

- Line 73 – you say the interview was a male qualitative cardiovascular researcher but then line 77 says four female science graduates conducted the interviews. Better explanation of everyone's role is needed.

We have revised text to clarify information about the interviewers and their role.

- If the topic guide was created based on the logic model, it would be useful to see the logic model – is this published elsewhere? If so, this should explicitly be referred to but if not, maybe think about adding it to the paper.

Yes, we have developed our topic guide based on logic model. We have previously published a figure that outlines the logic model in the protocol paper, which is also cited in the manuscript. (page no. 7, methods- in the manuscript).

Here's the citation to the protocol paper for your reference- Salam, A., Webster, R., Patel, A., Godamunne, P., Pathmeswaran, A., de Silva, H. A., ... & Laba, T. L. (2018). Process evaluation of a randomised controlled trial of a pharmacological strategy to improve hypertension control: protocol for a qualitative study. BMJ open, 8(8), e022317.

- Lines 97-99 – it sounds like 5 people in total were involved in the analysis, but who did what? How did this work in practice?

We have now revised text for clarity. Detailed description of contributions is given under 'Contributors'- on page 24.

- Was the MRC framework used in the analysis? If so how? It's not clear what kind of analysis was done – was it thematic, content etc. Did you use induction or deduction analysis etc.

Yes, MRC framework was used in the analysis. We have now added text for clarity.

Results

- Line 107 – what was the rationale and aim for including patients from the control/usual care group? This wasn't really mentioned in the methods.

To further understand context. This is a common practice in process evaluation. (6)

- Line 110 – how are research coordinators 'providers'? More needs explaining on this in the methods.

Nurses, pharmacists, and researchers based at the site and who were involved in the conduct of the trail were termed as providers.

- The average length of the interviews is concerningly low – how confident were you that you gathered all the necessary and relevant information? Definitely something to mention as a limitation.

We are confident enough that we have gathered all the necessary and relevant information. It is similar to other previously conducted studies with similar objectives. (6)

The interviews with the doctors were longer than those with the site coordinators, as the doctors were engaged in making healthcare decisions, whereas the site coordinators only provided information about trial activities, resulting in shorter interviews.

- Table 1 looks more like presenting baseline characteristics of a quantitative study. Would suggest changing to remove proportions and also provide information on patients and healthcare providers separately.

We have now removed proportions and provided information about the health care providers.

- It's not clear what exactly your themes are and whether there are sub-themes. Is 'healthcare setting' your first theme? A coding tree would be helpful and I suggest adding this in.

Themes	Contextual factors that underpinned trial outcomes	Implementation of the intervention and mechanisms of effect	Barriers and facilitators to implementing the triple pill strategy in clinical practice
Sub-themes	Screening, diagnosis, and awareness of hypertension	Better hypertension care within the trial	Availability of FDCs of BP-lowering medications
	Care pathway for patients with hypertension	Differential access to BP-lowering medications during the trial	Acceptability of the triple pill
	BP-lowering medication use pattern	Follow-up during the trial	Indication for, and limitations of, the triple pill
	Patient follow-up and modification of therapy	Simplification of BP-lowering therapy and improved efficacy with triple pill	Healthcare funding and provider education
	Access to BP-lowering medications	Differential assessment of adherence to BP-lowering medications	-
	Patients' adherence to BP-lowering medications	-	-

- Table 2 is not clear at all, I'm not sure what it's trying to say or refer to.

It compares usual care vs trial care vs routine practice.

- All direct quotes should be quotations

Changed

- Part of the results doesn't seem directly related to the processes of the trial – e.g. under 3.2.1 you talk about the process of screening but was this part of the trial? It's also not clear whether the information being reported is directly from the participants or is it an interpretation of the interview data/what participants said. This distinction is important. This all could be better understood with a clearer methods section.

Everything in the results was from the participants.

Discussion

- I feel the discussion is overall weak. It seems more to do with the trial results and not the results of the process evaluation. Can you provide recommendations based on your results for future implementation and scale up?

References

1. Vasileiou, K., Barnett, J., Thorpe, S. *et al.* Characterising and justifying sample size sufficiency in interview-based studies: systematic analysis of qualitative health research over a 15-year period. *BMC Med Res Methodol* **18**, 148 (2018). https://doi.org/10.1186/s12874-018-0594-7
2. Sanuade OA, Jacobson TA, Quintana A, Flowers FM, Abbasi H, Vu MH, Baldridge AS, Mejia J, Lazar D, Ciolino JD, Huffman MD, Kandula NR. Process Evaluation of a Double-Blind Randomized Controlled Trial to Assess the Efficacy and Safety of a Quadruple Ultra-Low-Dose Treatment for Hypertension Within a Federally Qualified Health Center Network (QUARTET USA). J Am Heart Assoc. 2024 Jan 2;13(1):e032236. doi: 10.1161/JAHA.123.032236. Epub 2023 Dec 29. PMID: 38156601; PMCID: PMC10863813.
3. Grant S, Hodgkinson J, Schwartz C, Bradburn P, Franssen M, Hobbs FR, Jowett S, McManus RJ, Greenfield S. Using mHealth for the management of hypertension in UK primary care: an embedded qualitative study of the TASMINH4 randomised controlled trial. Br J Gen Pract. 2019 Aug 29;69(686):e612-e620. doi: 10.3399/bjgp19X704585. PMID: 31262847; PMCID: PMC6607844.
4. Wei X, Deng S, Haldane V, Blacklock C, Zhang W, Zhang Z, Walley JD, King R, Hicks JP, Yin J, Zou G, Huang Y, Vergis M, Jun Z, Sun Q, Lin M. Understanding factors influencing antibiotic prescribing behaviour in rural China: a qualitative process evaluation of a cluster randomized controlled trial. J Health Serv Res Policy. 2020 Apr;25(2):94-103. doi: 10.1177/1355819619896588. Epub 2020 Jan 27. PMID: 31986910.
5. Allison Tong, Peter Sainsbury, Jonathan Craig, Consolidated criteria for reporting qualitative research (COREQ): a 32-item checklist for interviews and focus groups, International Journal for Quality in Health Care, Volume 19, Issue 6, December 2007, Pages 349–357, https://doi.org/10.1093/intqhc/mzm042

6. Mark D. Huffman,Abigail S. Baldridge,Danielle Lazar,Hiba Abbas,Jairo Mejia,Fallon M. Flowers,Adriana Quintana,Alema Jackson,Namratha R. Kandula,Donald M. Lloyd-Jones,Stephen D. Persell,Sadiya S. Khan,James J. Paparello,Aashima Chopra,Priya Tripathi,My H. Vu,Clara K. Chow,Jody D. Ciolino,Efficacy and safety of a four-drug, quarter-dose treatment for hypertension: the QUARTET USA randomized trial, Hypertension Research, 47, 6, (1668-1677), (2024).
